# Analyses of inter-individual variations of sperm DNA methylation and their potential implications in cattle

**DOI:** 10.1186/s12864-019-6228-6

**Published:** 2019-11-21

**Authors:** Shuli Liu, Lingzhao Fang, Yang Zhou, Daniel J.A. Santos, Ruidong Xiang, Hans D. Daetwyler, Amanda J. Chamberlain, John B. Cole, Cong-jun Li, Ying Yu, Li Ma, Shengli Zhang, George E. Liu

**Affiliations:** 1 College of Animal Science and Technology, China Agricultural University, Beijing, 100193 China; 2 USDA-ARS, Animal Genomics and Improvement Laboratory, Beltsville, MD 20705 USA; 3 Department of Animal and Avian Sciences, University of Maryland, College Park, MD 20742 USA; 4 Medical Research Council Human Genetics Unit at the Medical Research Council Institute of Genetics and Molecular Medicine, University of Edinburgh, Edinburgh, EH4 2XU UK; 5 Key Laboratory of Agricultural Animal Genetics, Breeding and Reproduction, Education Ministry of China, Huazhong Agricultural University, Wuhan, 430070 Hubei China; 6 Faculty of Veterinary & Agricultural Science, The University of Melbourne, Parkville, Victoria 3052 Australia; 7 Agriculture Victoria, AgriBio, Centre for AgriBiosciences, Bundoora, Victoria 3083 Australia; 8 School of Applied Systems Biology, La Trobe University, Bundoora, Victoria 3083 Australia

**Keywords:** Sperm DNA methylation, Methylation haplotype blocks, Variably methylated regions, Reproduction traits, Cattle

## Abstract

**Background:**

DNA methylation has been shown to be involved in many biological processes, including X chromosome inactivation in females, paternal genomic imprinting, and others.

**Results:**

Based on the correlation patterns of methylation levels of neighboring CpG sites among 28 sperm whole genome bisulfite sequencing (WGBS) data (486 × coverage), we obtained 31,272 methylation haplotype blocks (MHBs). Among them, we defined conserved methylated regions (CMRs), variably methylated regions (VMRs) and highly variably methylated regions (HVMRs) among individuals, and showed that HVMRs might play roles in transcriptional regulation and function in complex traits variation and adaptive evolution by integrating evidence from traditional and molecular quantitative trait loci (QTL), and selection signatures. Using a weighted correlation network analysis (WGCNA), we also detected a co-regulated module of HVMRs that was significantly associated with reproduction traits, and enriched for glycosyltransferase genes, which play critical roles in spermatogenesis and fertilization. Additionally, we identified 46 VMRs significantly associated with reproduction traits, nine of which were regulated by cis-SNPs, implying the possible intrinsic relationships among genomic variations, DNA methylation, and phenotypes. These significant VMRs were co-localized (± 10 kb) with genes related to sperm motility and reproduction, including *ZFP36L1*, *CRISP2* and *HGF*. We provided further evidence that rs109326022 within a predominant QTL on BTA18 might influence the reproduction traits through regulating the methylation level of nearby genes *JOSD2* and *ASPDH* in sperm.

**Conclusion:**

In summary, our results demonstrated associations of sperm DNA methylation with reproduction traits, highlighting the potential of epigenomic information in genomic improvement programs for cattle.

## Background

Emerging evidence shows that the sperm DNA methylome contributes to not only male fertility but also to early embryo development [1–4]. DNA methylation in sperm has been shown to be involved in many biological processes, including X chromosome inactivation in females, paternal genomic imprinting, silencing of transposable elements and DNA compaction [5–8]. Some alterations of sperm DNA methylation may persist into the early embryo and thus influence the transcriptome and epigenome in somatic tissues [9, 10], leading to variation in phenotypes of offspring [11]. In dairy cattle breeding, we use the breeding value (e.g., predicted transmitting ability, PTA) of a sire to measure his contribution to complex traits of offspring, after correcting for all known systematic effects. An elite bull often has thousands of daughters due to artificial insemination, yielding high reliable phenotypes (i.e., estimated breeding values). This offers a valuable source for understanding the relationships between sperm DNA methylation and complex traits in mammals, particularly in males.

Variation of DNA methylation among individuals has been speculated to affect susceptibility to complex diseases and resistance to drug treatment in human [12–14]. The epigenetic polymorphism, termed “variably methylated regions” or VMRs, were found to be enriched in various functional genomic features, like enhancers, CpG shores, 3’UTR etc., indicating their potential roles in transcriptional regulation [15, 16]. In addition, inter-individual methylation variations have been demonstrated to be associated with tissue-specific function and environmental adaptation [15]. For instance, VMRs within co-methylated networks in fibroblasts were enriched for four clusters of HOX genes. Furthermore, both genetic factors and environmental exposures like diet, stress, toxic exposure and exercise contribute to epigenetic variation [17–19]. Analysis of VMRs in human neonatal blood samples further indicated that VMRs were best explained mainly by either environmental factors and genotype interaction (GxE) or their additive effects (G + E) [20]. Additionally, SNPs involved in the significant GxE models were highly enriched with signals of genome wide association studies (GWAS) for complex diseases [20]. Studies have also revealed that, by targeting VMRs, the statistical power can be improved in epigenetic signature detection using epigenetic association studies (EWAS) [21]. While VMRs were studied in human and model organisms, to our knowledge, no studies have been published to investigate inter-individual variation of DNA methylation in cattle, particularly in sperm, which is of importance in dairy cattle due to the wide use of artificial insemination technology.

There are different methods to detect VMRs. In previous human studies, VMRs were determined by either merging the adjacent highly variable CpG sites within predefined windows [15, 22] or using the highly variable restriction enzyme (*Msp* I) fragments from reduced representation bisulfite sequencing. However, methylation levels of each CpG site within VMRs may be variable, raising a question of which CpG site to choose. For example, the most variable CpG site (tagCpG) within each VMR was often selected to represent it [15, 22]. This strategy may miss the information provided by other CpG sites. Some researchers directly utilized the single CpG site [21], which may be influenced by the technical noise and sensitivity in measuring single CpG methylation [23]. Because adjacent CpG sites tend to show the coordinated methylation statuses due to the progressivity of the DNA methylation and/or demethylation enzymes (DNMT1, DNMT3A/B and TET proteins), these co-methylated CpG sites may form methylation haplotypes, called methylation haplotype blocks (MHBs) [23]. A previous study showed that MHBs tend to be enriched in VMRs and functional regions [23]. Here, we hypothesized that the utilization of MHBs could improve the definition of VMRs by concentrating on biologically relevant regions, and enhance statistical power by reducing the multiple testing burden compared to the single-CpG method.

**Fig. 1:**
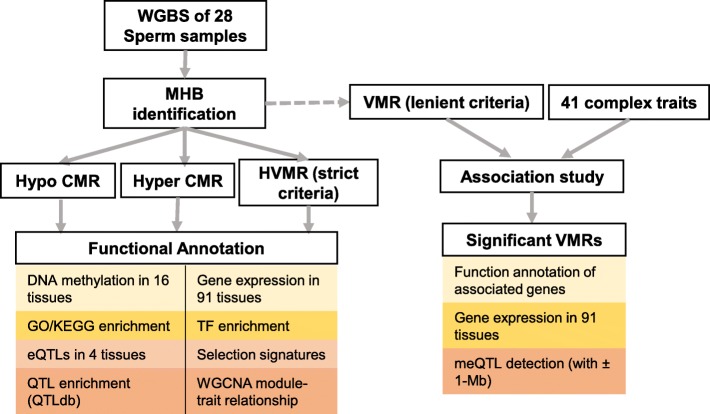
Schematic overview of the current study. We defined methylation haplotype blocks (MHBs) using whole genome bisulfite sequencing (WGBS) data of 28 sperm samples. We then detected the highly variably methylated regions (HVMRs), conserved hypomethylated regions (Hypo-CMRs) (average methylation level < 20%) and conserved hypermethylated regions (Hyper-CMRs) (average methylation level > 80%) based on the methylation variations among individuals. We next functionally annotated them by integrating DNA methylation, gene expression, GO/KEGG, transcriptional factor binding sites, QTL and WGCNA module-trait relationship. We further detected the variably methylated regions (VMRs) using lenient criteria. We associated the methylation levels of VMRs with 41 complex traits. We also annotated the significant VMRs by examining the functional annotation of their associated genes, and their corresponding expression across 91 tissues. We finally conducted cis-methylation QTL (± 1-Mb) analyses for significant VMRs

In this study, we functionally annotated the VMRs using a range of other information, including gene expression, predicted transcription factor binding sites, traditional quantitative trait loci (QTL), gene expression QTL and selection signatures (Fig. 1). We further associated VMRs with 41 complex traits and further explore the cis-SNPs that may regulate the detected significant epigenetic signatures within ±1-Mb. To our knowledge, this is the first attempt to reveal the potential impact of epigenomics on phenotypes on genome wide level.

## Results

### Identification and characterization of methylation haplotype blocks

Our correlation analysis of 28 WGBS data within various genomic elements, revealed that global methylation was highly conserved among individuals, i.e. Pearson correlations ranged from 0.914 to 0.995 (Additional file 1: Figure S1a). The top conserved genomic elements included 5′ UTRs, CG islands and promoters, while introns, 3’UTRs and exons were relatively dynamic among individuals.

**Fig. 2:**
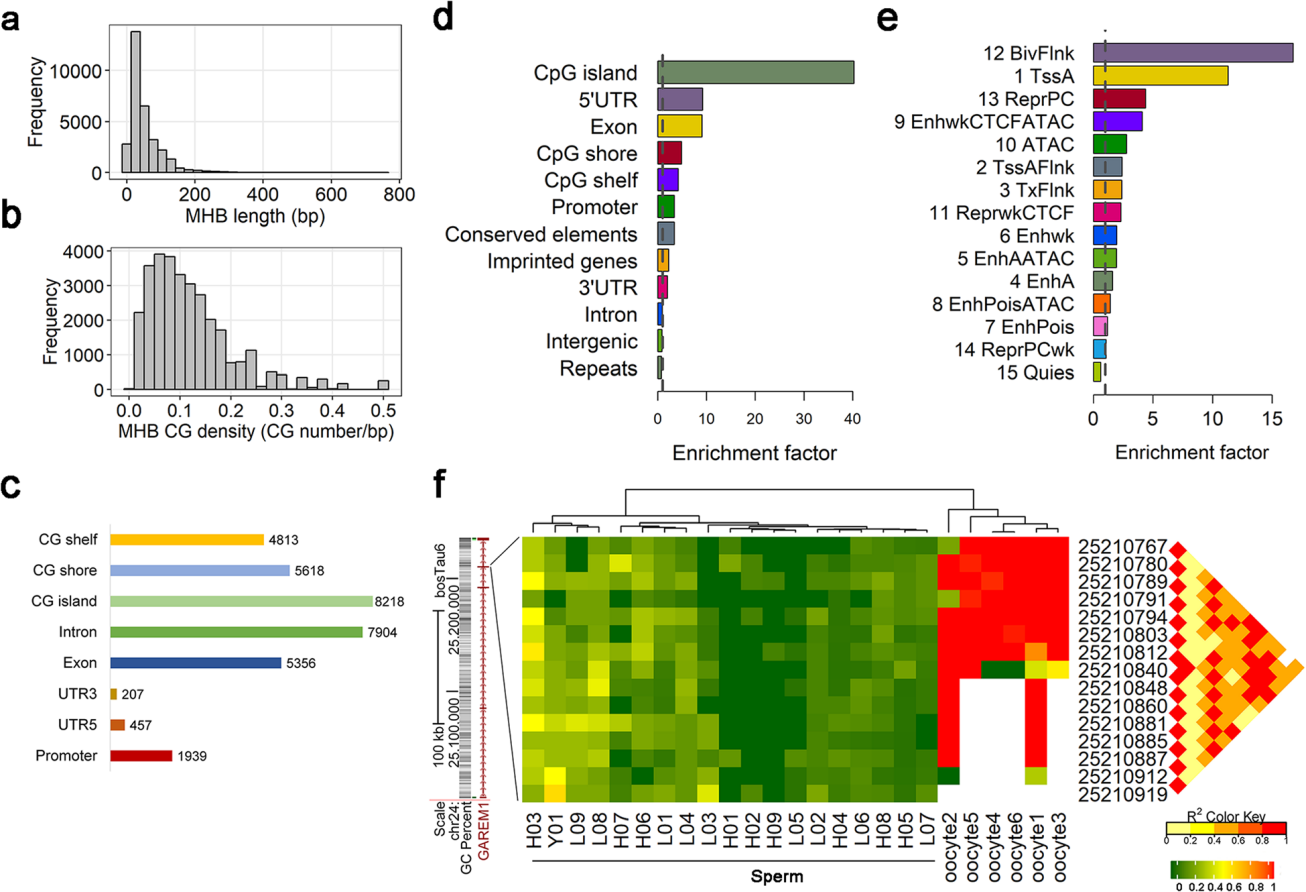
Characterization of sperm methylation haplotype blocks (MHBs) in cattle. **a** Length distribution of MHBs. **b** CG density (CG number per base pair) distribution of MHBs. **c** Co-localization of MHBs with known genomic elements. **d** Enrichment of MHBs in known genomic features. **e** Enrichment of MHBs in predicted chromatin core 15-states using chromHMM [25, 63]: 1 TssA: Active Tss; 2 TssAFlnk: Flanking active TSS; 3 TxFlnk: Transcrption at gene 5′ and 3′; 4 EnhA: Active enhancer; 5 EnhAATAC: Active enhancer & ATAC; 6 EnhWk: Weak active enhancer; 7 EnhPois: Poised enhancer; 8 EnhPoisATAC: Poised enhancer & ATAC; 9 EnhWkCTCFATAC: Weak enhancer & CTCF & ATAC; 10 ATAC: ATAC islands; 11 reprWkCTCF: Weak represeed CTCF; 12 BivFlnk: Flanking bivalent TSS/Enhancer; 13 ReprRC: Repressed Polycomb; 14 ReprPCWk: Weak repressed Polycomb; and 15 Quies: Quiescent/Low. **f** One example of MHB located in the exon 4 of predicted maternal imprinted gene *GAREM1*. Methylation levels of the MHB were low in sperm but high in oocyte

We calculated a pairwise “linkage disequilibrium” of CpG methylation (LD, *r*^*2*^) as previously reported [23], and partitioned the mappable genome (coverage > = 10; minimal size: 80 bp) into blocks using LD (*r*^*2*^) cutoff of 0.5 with at least 3 CpG sites within a block. We identified 31,272 MHBs (Additional file 2: Table S1) with an average size of 52 bp (Fig. 2a), and an average of 12 CpG sites per 100 bp (Fig. 2b). Pearson correlation analysis showed that methylation levels within MHBs were less correlated among individuals (0.52 ~ 0.86) compared to those of various genomic elements (Additional file 1: Figure S1b), which was concordant with the previous observation that MHBs were variable among individuals and highly enriched in VMR [15]. The MHBs also overlapped with multiple known genomic elements (Fig. 2c). Among all the MHBs, 64.6, 35.4% were located in intergenic regions or transcribed regions, respectively. The MHBs were highly enriched in CpG islands, 5’UTRs, exons and promoters (1000 times of permutation test using RegioneR [24], *P* < 0.001), indicating that they may play important roles in transcriptional regulation (Fig. 2d). Based on the 15 chromatin states in bovine rumen cells predicted using histone modifications (H3K4me3, H3K4me1, H3K27ac, H3K27me3) and other epigenome information (ATAC-seq and CTCF binding sites) [25], we observed MHBs had a significant enrichment for flanking bivalent TSS/enhancer (enrichment factor: 17) and active TSS i.e. promoters (enrichment factor: 11) (Fig. 2e). We also observed an enrichment of MHBs in imprinted genes (enrichment factor: 2.22) like HOXA genes, *IGF2*, and *IGF2R*. One example is the methylation block containing 15 CpG sites in exon 4 of a predicted maternal imprinted gene *GAREM1* (Fig. 2f). We observed distinct methylation patterns of this region between sperm and oocytes [26], where sperm were consistently lowly methylated while oocytes were highly methylated. Collectively, blocks with coordinately methylated 5mC were likely to capture the epigenetic signatures associated with transcriptional regulation.

To explore the conservation of MHBs among species, we compared the detected MHBs between cattle and human by converting the MHB coordinates to the human hg19 genome using the liftOver tool in the UCSC browser. It is noted that human MHBs were predicted using human somatic tissues instead of sperm, which were published before [23]. Out of all the bovine MHBs, 51.8% were successfully converted with the minimal match of 0.8. A total of 1952 bovine MHBs were overlapped with those from human [23], and associated with 1701 human genes, while the cattle-specific MHBs were associated with 5832 genes. Interestingly, genes overlapping with common MHBs between human and cattle (HCMHBs) showed an enrichment of GO terms related to early embryonic development, while those associated with cattle-specific MHBs (CMHBs) were mainly enriched in the development of nervous system (FDR < 0.05, Additional file 1: Figure S2a). We also found HCMHBs overlapped with more genes (Additional file 1: Figure S2b) and were more conserved among mammals (indicated as the PhastCon scores, https://genome.ucsc.edu/goldenPath/help/phastCons.html) than CMHBs (Additional file 1: Figure S2c). Further study is required to understand the biological mechanisms underlying the evolution of MHBs among species.

### Inter-individual variation and conservation in sperm DNA methylome

**Fig. 3:**
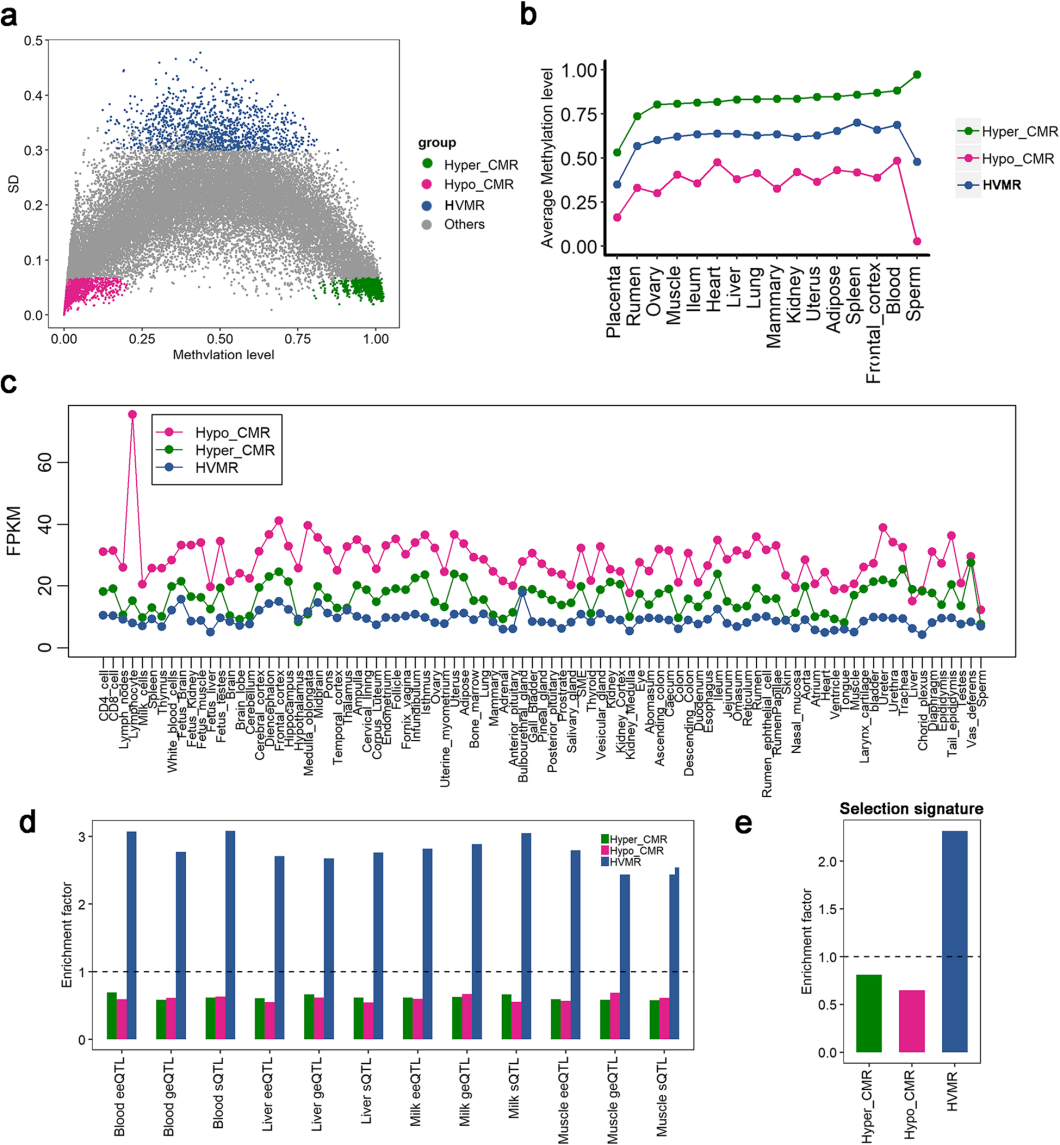
Comparison of three categories of regions with extreme methylation variation in sperm. **a** Standard deviation (SD) vs. mean sperm DNA methylation of all methylation haplotype blocks (MHBs), and distribution of highly variable methylated regions (HVMRs), hypomethylated conserved regions (Hypo-CMRs) and hypermethylated conserved regions (Hyper-CMRs). **b** Average methylation levels of HVMR, Hypo-CMRs and Hyper-CMRs in 16 somatic tissues. **c** Average expression levels (FPKM value) of genes associated with HVMR, Hypo-CMRs and Hyper-CMRs in 91 tissues and cell types. **d** Enrichments of three categories of expression QTL in HVMR, Hypo-CMRs and Hyper-CMRs (eeQTL: exon expression QTLs; geQTL: gene expression QTLs; sQTL: splicing QTLs; these expression QTLs were detected from dairy cattle blood and milk cells, liver and muscle [28]). **e** Enrichments of selection signatures differentiating dairy and beef cattle breeds [30] in HVMR, Hypo-CMRs and Hyper-CMRs

MHBs were previously shown to have an extremely high enrichment in VMRs [23]. To characterize the methylation dynamics among individual bulls, we defined three categories of regions from MHBs in terms of their methylation variations (See Methods), 1) highly variable methylation regions (HVMRs, *n* = 1681) with extremely high methylation variations, 2) conserved hypomethylated regions (hypo-CMRs, Average methylation level < =0.2, *n* = 3371), 3) conserved hypermethylated regions (hyper-CMRs, Average methylation level > =0.8, *n* = 1594) (Additional file 2: Table S2). As expected, moderately methylated MHBs were more likely to be variable among individuals than highly or lowly methylated MHBs (Fig. 3a). We further observed that methylation differences between HVMRs and CMRs persist into multiple somatic tissues, such as rumen, ovary and placenta (Fig. 3b). We collected bovine transcriptomes of 91 tissues/cells from published data of previous studies. Similarly, expression differences between HVMR- and CMR-associated genes (genes overlapped with MHB regions in promoters and gene bodies) were also consistent in 91 bovine tissues/cells. Genes associated with hypo-CMRs generally had the highest expression, followed by genes associated with hyper-CMRs and genes associated with HVMRs (Fig. 3c). Functional annotation further revealed that genes associated with hypo-CMRs were engaged in basic cell-function, including transcription, DNA binding and nuclear chromatin (Additional file 1: Figure S3a), suggesting that these genes were mainly house-keeping genes. For example, Hypo-CMRs were enriched for developmental motifs, like HOXD13, and motifs involved in cell proliferation and differentiation, like MYB, KHDRBS2 and SRSF10. Hyper-CMRs were enriched in motifs associated with hemopoietic development and alternative splicing, like MZF1 and CELF2. In contrast, we speculated that HVMRs may be more likely to harbor tissue-specific expressed genes. Furthermore, our transcription factor binding motif analysis validated that the HVMRs were enriched for motifs with divergent functions, such as ZNF711 for cognitive disability, PBX3 for leukemia and PKNOX1 for adult spermatogenesis [27] (Additional file 1: Figure S3b). Additionally, human orthologous genes in HVMRs were also enriched in tissue-specific expressed genes (enrichment factor: 1.84; *P* = 5.52 × 10^−6^; Fisher’s exact test), while human orthologous genes in hypo-CMRs and hyper-CMRs were enriched in house-keeping functions (enrichment factor: 1.49 and 1.64; *P* = 7.01 × 10^−8^ and 6.86 × 10^−7^) (Additional file 1: Figure S3c). We further overlapped HVMRs, hypo- and hyper-CMRs with multiple types of expression QTLs, including splicing QTLs (sQTLs), gene expression QTLs (geQTLs) and exon expression QTLs (eeQTLs), from previous analyses [28, 29]. All three types of QTLs were highly enriched in HVMRs (*P* < 2.2 × 10^−16^, Fisher’s exact test), but depleted in hypo-CMRs and hyper-CMRs (*P* < 2.2 × 10^−16^) (Fig. 3d), which were consistent among all four tissues (muscle, liver, blood and milk cells). This result indicates that genomic regions associated with sperm HVMRs could play key roles in regulation of gene expression and splicing among individuals. We further found an enrichment of HVMRs (*P* < 2.2 × 10^−16^) in selection signatures with higher frequency in dairy rather than in beef breeds [30], suggesting that HVMRs might play roles in positive selection and adaptive evolution (Fig. 3e). Collectively, our data revealed distinct DNA methylation variation patterns in sperm might influence the transcriptional regulation and evolution.

### HVMRs were associated with QTLs of reproduction traits

**Fig. 4:**
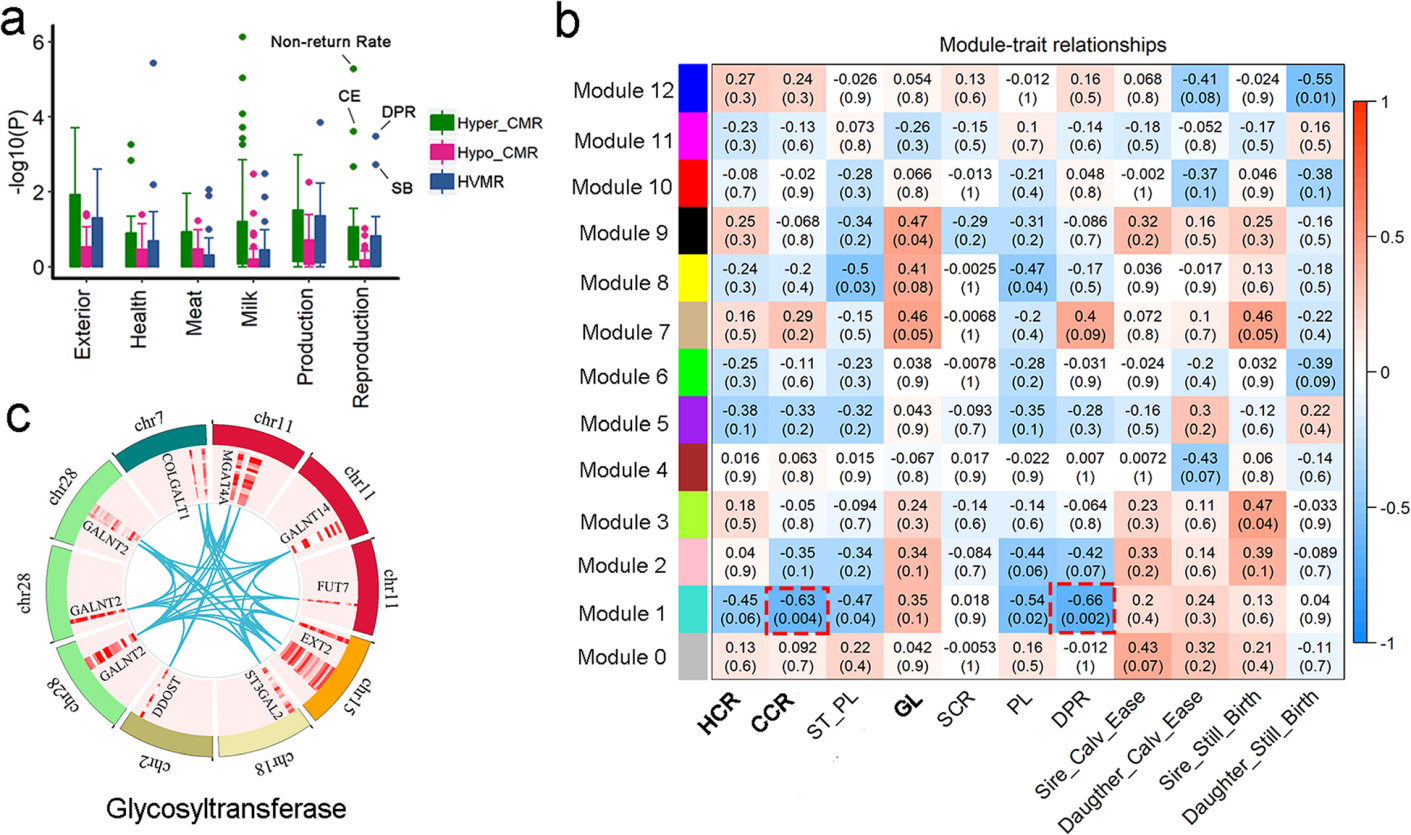
Relationship between methylation variations in sperm and complex traits. **a** Enrichments of six QTL categories (including 232 traits) from Cattle QTL database in HVMRs, Hypo-CMRs and Hyper-CMRs. CE: calving ease; DPR: daughter pregnancy rate; SB: still birth. **b** Module-trait relationships using a weighted correlation network analysis (WGCNA) (Only reproduction traits were tested). Elements in red dash box were two traits most significantly associated with module 1. HCR: heifer conception rate; CCR: cow conception rate; ST_PL: standard length of productive life; GL: gestation length; SCR: sire conception rate; PL: length of productive life; DPR: daughter pregnancy rate. **c** Co-methylated regions with 15 MHBs in module 1 enriched for glycosyltransferase genes. M1, M2 and M3 represents three MHBs located with the *GALNT2* gene

To explore the relationship between HVMRs in sperm and complex traits, we first examined the QTL regions of six categories of traits (*n* = 232) (Exterior, health, Meat, milk, production and reproduction traits) from the Cattle QTL database (https://www.animalgenome.org/cgi-bin/QTLdb/BT/index). We observed that both hyper-CMRs and HVMRs had higher enrichments for QTL signals of complex traits than hypo-CMRs. Of note, HVMRs tend to be specifically and significantly enriched for QTL signals of reproduction traits, with the top associated traits were daughter pregnancy rate (DPR; FDR = 0.03) and stillbirth (SB; FDR = 0.1). While hyper-CMRs were highly associated with a range of complex traits such as milk production traits, non-return rate and calving ease (CE) (Fig. 4a).

To further investigate the relationship between HVMRs and reproduction traits, we grouped the co-methylated HVMRs into 12 distinct modules using WGCNA analysis [31], and associated each module with 11 reproduction traits in our 19 bulls of similar age (1~2-year-old), after correcting for the genetic relatedness (Fig. 4b). We observed Module 1 was significantly (*P* < 0.05) negatively correlated with several reproduction traits, including heifer conception rate (HCR), cow conception rate (CCR), standard length of productive life (ST_PL), length of productive life (PL), and DPR, but positively correlated with gestation length (GL) and sire still birth. This was consistent with their negative genetic correlations as previously reported [4]. Interestingly, the 15 co-methylated MHBs in Module 1 were enriched for glycosyltransferase genes (FDR = 0.0046) (Fig. 4c). Although the exact mechanisms remain elusive, published results reported that glycosyltransferases were the main enzymes in glycosylation and responsible for the synthesis of glycans which play pivotal roles in spermatogenesis [32, 33]. Previous studies have shown that glycosyltransferases are crucial for spermatozoa maturation in epididymis and sperm survival in the female reproductive tract [34, 35]. Sperm glycans continue to be modified by glycosyltransferases and carry out functions in female reproductive tracts like acrosome reaction, protection from innate and adaptive female immunity and passage through the cervical mucus [34]. Additionally, glycosyltransferase activities are potentially involved in modification of the glycan on the zona pellucida that boost the its ability to bind spermatozoa [36].

### Association analyses between VMRs and reproduction traits

**Fig. 5:**
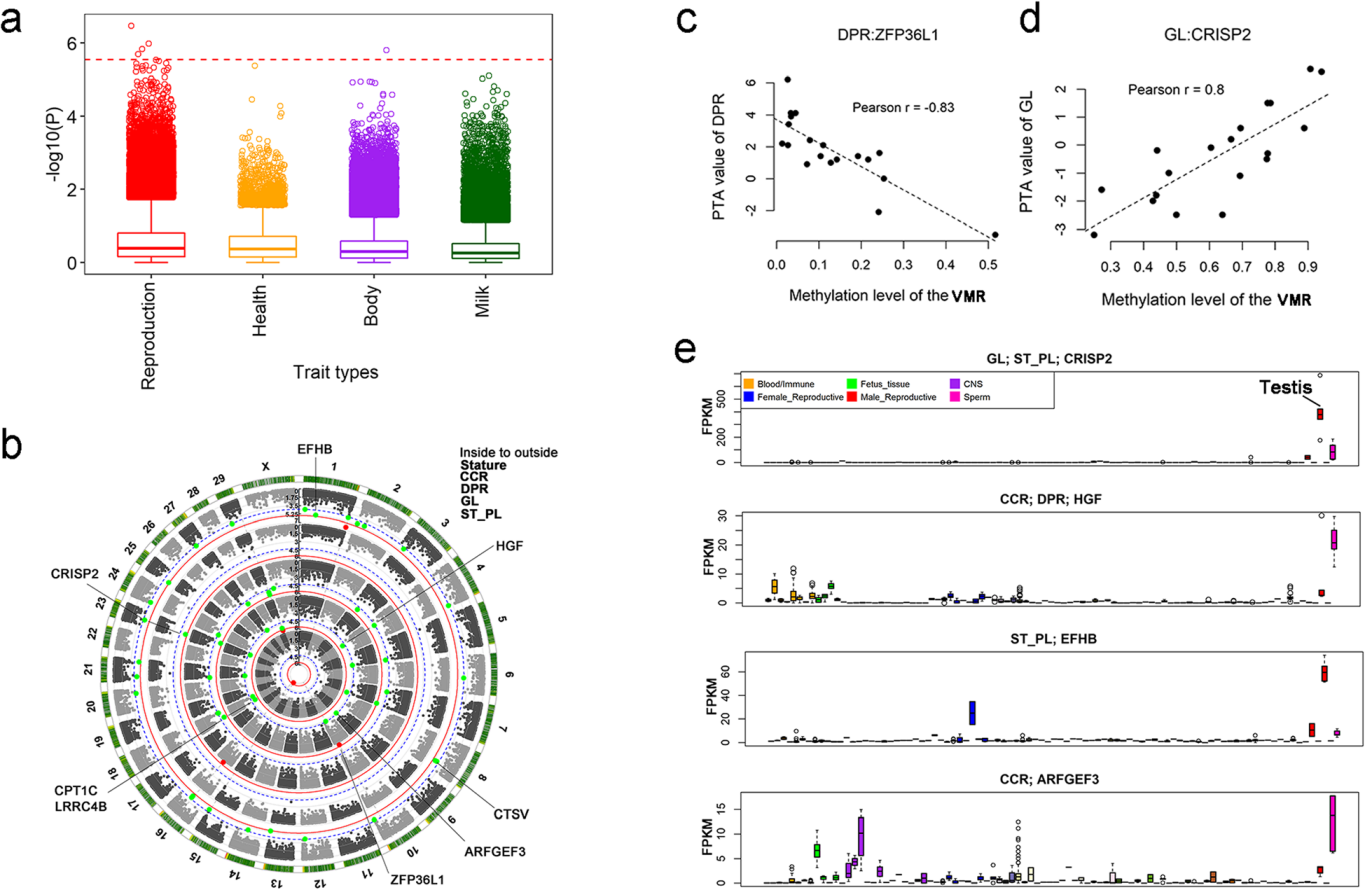
Associations between variably methylated regions (VMRs) and reproduction traits. **a** The -log(*P*) values of associations of VMRs with four types of complex traits. Red dot line indicates the significant *P* value after Bonferroni correction (*P* < 2.89 × 10^-6^; 0.05/17323). **b** Manhattan plots of five traits with significant/suggestively significant VMRs. Red dots indicated the significant VMRs (*P* < 2.89 × 10^−6^; 0.05/17323). Green dots indicated the suggestive significant VMRs (*P* < 5.77 × 10^−5^; 1/17323). CCR: cow conception rate; DPR: daughter pregnancy rate; GL: gestation length; ST_PL: standard length of productive life. **c** Correlation between predicted transmitting ability (PTA) values of DPR and methylation levels of VMR within the *ZFP36L1* gene. **d** Correlation between PTA values of DPR and methylation levels of VMR located downstream of *CRISP2* gene. **e** Expression levels (FPKM values) of four genes associated with significant VMRs across 91 bovine tissues

To further explore the association of sperm methylation with complex traits, we tested the association of VMRs with 41 complex traits individually using 19 samples from bulls with similar ages (1 to 2-year-old). Unlike HVMRs, VMRs were defined using a less strict criteria (the *p* value of chi-square test less than 1 × 10^− 4^) to avoid missing important signals (See Methods). This resulted in 17,323 VMRs for subsequent analysis, accounting for 55.4% of all identified MHBs. Methylation levels of VMRs were transferred from β values to M values for association tests [37]. We found that VMRs were more likely to be associated with reproduction traits than other traits (Fig. 5a). After Bonferroni correction, we detected 5 significant (*P* < 2.89 × 10^− 6^, 0.05/17323) VMRs and 41 suggestively significant VMRs for five traits (*P* < 5.77 × 10^− 5^, 1/17323), of which four were reproduction traits, including DPR, CCR, GL, and ST_PL, and the remaining one was related to stature. (Fig. 5b, Additional file 2: Table S3). The results were consistent with the above mentioned WGCNA results. About half (*n* = 25) of these detected VMRs were within 10 kb of annotated genes, and some of them had known functions in male or female fertility. The most significant VMR associated with DPR was within exon 2 of the *ZFP36L1* gene. We observed a highly negative correlation (Pearson *r* = − 0.83) between methylation levels of this VMR and DPR (Fig. 5c). The *ZNF36L1* gene is crucial for female fertility, and the disruption of this gene will result in embryo lethality [38]. Another example was the suggestively significant VMR associated with GL, which is located ~ 5.9 kb downstream of the *CRISP2* gene. We observed a positive correlation (Pearson *r* = 0.8) between methylation levels of this VMR and GL (Fig. 5d). Although the mechanisms are not currently understood, existing literature demonstrated that CRISP2 is a testicular sperm protein involved in spermatogenesis and it participates in acrosome reaction and gamete fusion [39–41]. We examined the transcriptome of 91 bovine tissues, and observed four genes associated with significant VMRs (*CRISP2*, *HGF*, *EFHB* and *ARFGEF3*) showing high expression patterns almost exclusively in sperm and testis (Fig. 5e).

### Examples of the trait-associated VMRs which were influenced by genetic variations

**Fig. 6:**
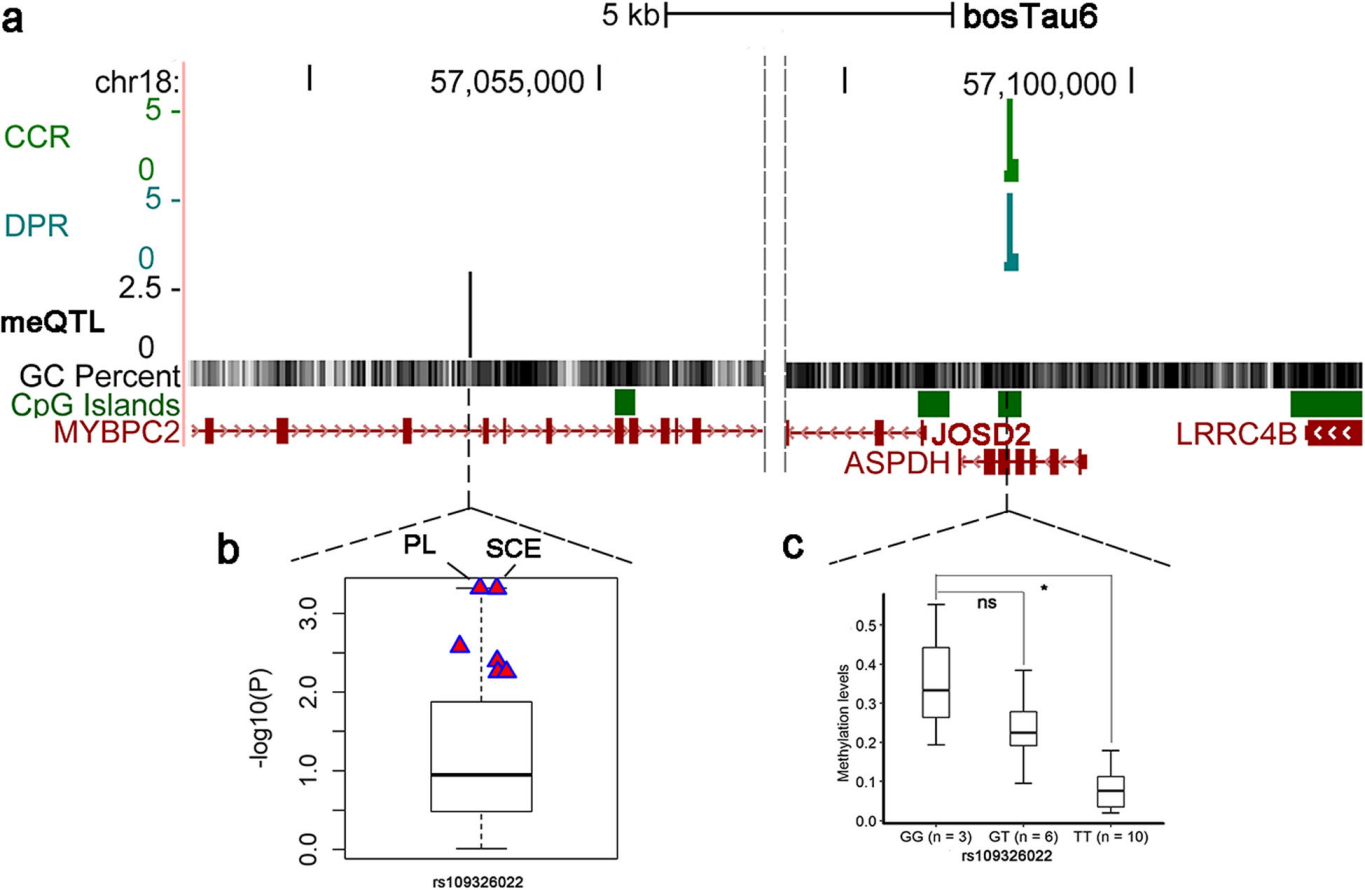
A trait-related variably methylated region (VMR) was associated with a SNP within 1 Mb distance. **a** UCSC browser of the VMR associated with CCR and DPR as well as the methylation QTL (meQTL) associated with the trait-related VMR (chr18: 57097832–57,097,893). Y axis indicates the –log10(*P*) from association test. **b** Association studies between the meQTL (rs109326022) and 35 bovine complex traits in 27, 214 Holstein bulls [44]. PL and SCE were the most significant traits associated with the meQTL. **c** Methylation levels of the trait-related VMR in three genotypes of rs109326022

To dissect the impact of genetic variations on trait-associated VMRs, we obtained 80 K SNPs genotypes for the 19 individuals of similar age being studied. Using an R package MatrixEQTL [42], we tested SNPs within 1 Mb (59 SNPs on average tested for each VMR) surrounding the detected significant or suggestively significant VMRs. Out of the 46 VMRs, nine were associated with at least one cis-SNP (FDR < 0.2). By overlapping these significant methylation QTLs (meQTLs) with the Cattle QTL database, we observed that meQTLs of three trait-associated VMRs were located within QTLs of reproduction traits (conception rate, still birth, calving ease, DPR, PL etc.). An interesting example is a trait-associated VMR (chr18: 57097832–57,097,893) located in the exon 5 of *ASPDH* and upstream (1.5 Kb) of *JOSD2* (Fig. 6a). rs109326022 is the most significant SNP associated with the VMR methylation level. Among the three genotypes, individuals with GG have the highest DNA methylation level but lowest PTA values for DPR and CCR, and those with TT have the lowest DNA methylation level but highest PTA values (Fig. 6c). This cis-meQTL was located within QTLs of PL, calving ease, still birth and calf size from Cattle QTL database. It was also detected as an eeQTL for *JOSD2* in blood, liver and milk cells [30]. On the other hand, a previous DNA methylation study identified *ASPDH* as a gene enriched in low-fertility sires [43]. To further validate the SNP effects on a range of complex traits, we examined the associations of rs109326022 with 35 complex traits in 27, 214 Holstein bulls [44]. This cis-meQTL was most significantly associated with PL and SCE among all tested traits (Fig. 6b), which might indicate the co-regulatory, intrinsic relationships among the cis-meQTL, sperm DNA methylation, and fertility traits. However, this cis-meQTL did not reach genome-wide significance for PL and SCE in the single-marker GWAS due to the very strict threshold and its small effect.

## Discussion

To our knowledge, this is the first report to characterize the inter-individual variation of sperm DNA methylation and to explore their associations with complex traits in cattle. We demonstrated that HVMRs were distinct from CMRs in terms of methylation levels, expression patterns and their association with complex traits. HVMRs may associate with tissue-specific gene expression and play roles in transcriptional regulation. Our results showed that the sperm epigenetic variations were associated with reproduction traits in cattle.

In our association studies, about half of the significant/suggestively significant VMRs were within 10 kb of annotated genes (Fig. 5b). Some of these genes were functionally important in reproduction. Besides *ZFP36L1* and *CRISP2* mentioned before, we detected the *HGF* gene, whose VMR was significantly associated with DPR. The VMR was located about 4400 bp downstream of *HGF*. Within 91 collected tissues, we found that *HGF* was specifically highly expressed in sperm. A previous study has shown that *HGF* is expressed by Sertoli cells and active during all phases of prenatal and postnatal testis development [45]. HGF and its only acceptor c-Met are involved in testis and ovary differentiation. HGF also has a role in mediating the spermatogenesis and sperm quality in different aspects [46, 47]. The level of HGF was reported to be changed in a comparison between healthy and infertility individuals [48]. In addition, we found one of our significant VMRs (chr18: 57097832–57,097,893) was also located within a differentially methylated region (DMR) (chr18: 57097376–57,098,221) in previous analysis by comparing bulls with high and low male fertility [43]. This significant VMR was identified to associate with CCR and DPR simultaneously. We also identified another nearby VMR (chr18: 56560453–56,560,476) associated with DPR in BTA18 (Additional file 1: Figure S4). These VMRs were located within a very prominent QTL for various fertility traits on chromosome 18 (BTA18, roughly located between 50 Mb and 60 Mb) [49]. This was also supported by our previous study, which reported sperm DNA methylation alterations in this region were associated GL, sire conception rate (SCR), body depth (BDE), and CCR [4]. Therefore, the influence of the prominent QTL on fertility traits could be a combination of both genetic and epigenetic mechanisms.

Our results indicated the sperm DNA methylation variation associates with reproduction traits in bulls, which were estimated based on the reproduction performance of their daughters, such as DPR, CCR, GL and PL. There are two potential mechanisms could lead to these associations. One is the transgenerational inheritance of sperm epigenome. While DNA methylation erasure occurred after fertilization, induced transgenerational epimutations appear to be protected from it. For example, researchers have shown that male mice fed with unbalanced diets could lead to the metabolic disease in the offspring, coupled with sperm epigenetics alteration [10, 50, 51]. Sperm DNA methylation and sperm RNA could both persist into embryo and even adult tissues, impacting the phenotypes of offspring. Another potential mechanism is that the genetic factors, such as the genetic variants like SNPs associated with DNA methylation (meQTLs), are transmitted to offspring via sperm DNA. We have shown that the genetic effects on DNA methylation could not be dismissed, as about 20% of trait-associated VMRs were influenced by genetic variations. A monozygous and heterozygous twin study reported that contribution of additive genetic factors on methylation variable sites were 23.0% on average [52]. Another study demonstrated that 44% of the methylation variation was best explained by genetic factors [20]. Additionally, meQTLs were previously suggested to be conserved among tissue types and developmental stages [53, 54]. Therefore, although our study performed an initial exploration between sperm DNA methylation and sire fertility traits, it will be interesting for future work to study the mechanisms how sire sperm epigenetic signatures may impact reproduction traits in female progenies. For example, comparing epigenetic information between sire sperm and embryo could provide insights on retained DNA methylation marks.

In our study, we identified VMRs based on MHBs, which were robust and sensitive [23]. Focusing on MHBs helped us to narrow the scope and improve the statistical power for subsequent methylation association analyses. Especially, we found that MHBs were enriched in functionally important elements, like CpG islands, gene promoters, and imprinted genes, which were in concordance with previous characterization of MHBs in human and mice [23, 55]. We revealed extreme enrichment of MHBs in flanking bivalent TSS/enhancer (BivFlnk) and active TSS (TssA) using annotated chromatin states in rumen epithelial cells. BivFlnk colocalizes both active (H3K4me3) and repressive (H3K27me3) histone modifications, and associates with genes of developmental importance [56]. TssA are active promoters and are associated with developmentally important genes [56]. Functional regions like active promoters and bivalent TSS were stable among cell types or tissues [57, 58]. Because of their conservation among tissues, it is possible to roughly annotate the MHBs in sperm using chromatin states in rumen epithelial cells.

Reproduction traits are complex and influenced by both genetic and epigenetic factors. Distinct from human and mouse, because of artificial insemination, we can measure the paternal contribution of sires to their offspring highly reliably. One advantage of our study was the high reliability of phenotype. The mean reliability for PTA estimation of GL, DPR, CCR, ST_PL and Stature was higher than 88%. Also we used sperm as our target to analyze the correlation between DNA methylation and phenotypes. Sperm is the only vector to deliver paternal genetic and epigenetic information to offspring, contributing to the variation of phenotypes in offspring (like female reproduction traits and milk traits). In our study, we also selected individuals with lowest relatedness and controlled their ages to around 1~2-year-old to limit confounding factors.

One limitation of our study is the small sample size (*n* = 19) to explore the preliminary associations between VMRs and complex traits. Even though WGBS costs less than before and has become more prevalent, it is still unaffordable to have a large number of samples tested. Therefore, in the near future it is urgent to design either PCR- or array-based high-throughput DNA methylation assays, for example, a low-density bovine methylation array covering important functional regions similar to the Infinium human methylation arrays. This would allow for the confirmation of our findings in a larger sample size.

## Conclusions

The significant enrichment of QTLs, eQTLs and selection signatures in HVMRs indicated the potential roles of methylation variation in sperm on transcriptional regulation, as well as complex trait and adaptive evolution. Our preliminary co-methylation analysis and methylation association study also suggested the potential impacts of sperm methylation variation on reproduction traits, despite their elusive natures. Additionally, we found that the effect of a prominent QTL region on BTA18 on female fertility traits could be related to both genetic and epigenetic mechanisms. In summary, our study of sperm DNA methylation variation provides novel insights into the biological basis underlying complex traits in cattle, and supplies valuable epigenetic hypotheses for future explorations.

## Methods

### Data processing and methylation haplotype blocks detection

A total of 28 sperm samples were used, and their age and coverage are listed in Additional file 2: Table S4. Twenty-six sperm samples were collected from 23 (1 to 5-year-old) fertile, health and representative U.S. Holstein AI bulls, and the other two sperm samples were collected from two 7-year-old Chinese Holstein AI bulls. All of semen samples passed QC tests (including microscopic examination of sperm count, motility, and abnormality, other laboratory tests) to qualify for commercial distributions. Semen samples were collected from bulls using a standardized procedure with artificial vaginas. Genomic DNA was isolated according to the QIAamp DNA MiniKit protocol (QIAGEN, Valencia, CA, USA). DNA quality was assessed using the 2100 Bioanalyzer (Agilent Technologies, Santa Clara, CA, USA) and spectrophotometer (NanoDrop Technologies, Rockland, DE) for DNA concentration, degradation, purify (OD260/280) and potential contamination. The qualified genomic DNA from sperm samples were used to construct libraries as described in Zhou et al. [57]. Programs FastQC v 0.11.2 and Trim Galore v 0.4.0 were used to obtain the read quality and filter the sequences, respectively. Clean reads were subsequently mapped to the reference genome (UMD3.1) using bowtie2 under the Bismark software (0.14.5) with default parameters. The methylcytosine information was extracted using bismark_methylation_extractor after deduplicating the duplicated reads.

We identified the MHBs using MONOD2 as described [23]. Briefly, we split the bovine UMD3.1 genome into non-overlapping “sequenceable and mappable” segments (mean size: 2.9 Mb and total size: 2.63Gb) using the combined WGBS data from 28 sperm samples. The mapped reads were converted into methylation haplotypes within each mappable segment. We then calculated MHBs based on the correlation patterns of methylation levels of neighboring CpG sites. Candidate MHBs were defined as the genome regions in which the r^2^ value of two adjacent CpG sites was no less than 0.5. MHBs with more than 2 CpG sites were kept for subsequent analysis.

### Enrichment analysis of methylation haplotype blocks for functional genomic regions

Genomic elements such as exons, introns, 5’UTRs, 3’UTRs and gene bodies were downloaded from Ensembl. Repeats and CpG islands were collected using the UCSC Table browser. Promoters were defined as the regions from upstream − 2 kb of TSS to TSS. Fifteen chromatin states were estimated using 4 histone marks (H3K4me3, H3K4me1, H3K27ac, H3K27me3), ATAC-seq and CTCF-seq from rumen epithelial primary cells in our previous study [25]. Fifteen chromatin states are active TSS, flanking active TSS, strong transcription, weak transcription, enhancers, bivalent enhancer etc., as shown in Roadmap project (https://egg2.wustl.edu/roadmap/web_portal/chr_state_learning.html#core_15state). Enrichment analysis was performed using R package regioneR [24] (Permutation test: 1000) and the mappable segments were used as the background. Expression QTL data including sQTL, geQTL and eeQTL in blood and milk cells, liver and muscle were retrieved from the previous study [28]. The summary data of selection signatures differentiating dairy and beef cattle were obtained from [30] using the 1000 Bull Genomes Project data (Run6) [59]. All the genome coordinates were based on UMD3.1/btau6.

### Identification of highly variable methylated regions and conserved methylated regions

Average methylation level of MHBs were calculated using a weighted methylation level method as described [60]. Methylation levels of regions which didn’t meet 5 × coverage for CpG sites were assigned to “NA”. We filtered out the MHBs where more than 13 individuals had methylation levels of “NA”. After filtering, 29,542 MHBs were kept for subsequent analysis. We then identified the HVMRs by overlapping the results of standard deviation (SD)-based method and chi-square test method. For the SD-based method, we firstly calculated the median SD for the MHBs. We then compared the SD of the methylation levels of each MHB to the median SD using the chi-square test for variance. We used a significant threshold of 0.01. To control for the family-wise error rate, MHBs with a *P* ≤ 3.39 × 10^−7^ (Bonferroni corrected) and SD larger than median level were identified as candidate HVMRs. For the chi-square test method, we treated each CpG result (methylated or unmethylated) in an MHB as an individual observation as previously described [16]. Candidate HVMRs were identified using the threshold of *P* ≤ 3.39 × 10^−7^. Regions identified by both above methods were termed HVMRs. Hypo-CMRs were regions identified using SD method, of which *P* ≤ 3.39 × 10^−7^, SD was smaller than median level and average methylation levels among individuals ≤0.2. Hyper-CMRs were regions identified using SD method, of which *P* ≤ 3.39 × 10^−7^, SD was smaller than median level and average methylation levels among individuals ≥0.8.

### Methylation association studies

We collected the individuals (*n* = 19) with similar ages (1~2-year-old) to process the methylation association studies (Additional file 2: Table S4). We found the methylation levels of MHBs tend to be dynamic among individuals. To increase the power of the association studies, we conducted two steps of quality control. We first filtered out the MHBs (*n* = 9,331) relatively conserved among individuals (*P* > 3.39 × 10^-9^, Bonferroni corrected) using the chi-square test method. Then we excluded the MHBs (*n* = 2,888) of which methylation levels in 19 individuals had NA values. After filtering, we called these inter-individual variable MHBs as VMRs. Finally, we obtained 17,323 VMRs for association studies. Since average methylation levels can be skewed, to avoid undue influence from outliers, we transferred them to the M-value using the β-value to M-value method [37]. We also collected the phenotypes of 41 bovine complex traits (PTA values of daughter’s traits and EBV values of sire conception rate) for the individuals. The statistics summary for these traits are shown in Additional file 2: Table S5. Detailed trait description and trait measurements can be found at https://www.uscdcb.com/. To adjust the relationship among the individuals, we conducted the principle component analysis (PCA) using genotype data of 80 K SNP array. We included the first two components (PC1 and PC2) into our association analyses. We used linear regression models: y = PC1 + PC2 + Meth; where y is the PTA values/estimated breeding value of 41 traits, Meth is the M-value of VMRs. After Bonferroni correction, we reported significant VMRs (*P* < 2.89 × 10^−6^; 0.05/17323) and suggestive significant VMRs (*P* < 5.77 × 10^−5^; 1/17323). All analyses were performed using R (3.5.3).

### Gene expression across 91 bovine tissues

We obtained 723 transcriptomes of 91 bovine tissues, of which 567 were collected from NCBI SRA/GEO databases and 156 samples were generated locally. Accessions for all datasets were SRP042639, GSE41637, SRP102212, SRP122763, SRP067373, SRP111067, GSE108840, GSE74076, ERP109534, GSE63509, SRP136662, GSE131849, GSE128075 and GSE129416.

### Identification of cis-meQTL

We tested the SNPs within 1 Mb distance from VMRs in association with methylation. A total of 79,294 probes from 19 individuals were processed. After filtering out the probes with only two genotypes and one of them contained less than 3 individuals, we kept 68,921 probes for subsequent analysis. Methylation levels of VMRs were transferred from β values to M values. We performed association analyses between cis-SNPs and M values of VMRs using MatrixEQTL package [42]. SNPs with FDR < = 0.2 were significant cis-meQTL for target VMRs.

### Other downstream bioinformatics analysis

We conducted gene functional annotation enrichments using online software, DAVID v6.8 [61]. We used HOMER [62] for motif discovery and prediction of TF binding sites considering the MHB as background. We conducted QTL enrichment analyses, with a hypergeometric test, for hypo- and hyper-CMRs and HVMRs by using cattle QTLdb (Release 37, Dec. 23, 2018). We arbitrarily considered genes overlapping or closest to the lead SNP in each QTL as the candidate genes for a trait. We only chose complex traits with more than five candidate genes to perform the enrichment analysis. *P*-value were adjusted using the FDR method.

## Supplementary information

**Additional file 1: Figure S1.** Correlation among individuals. (a) Pearson correlation among individuals of known genome features. (b) Heatmap of Pearson correlation in methylation haplotype blocks (MHBs) among individuals. **Figure S2.** Comparison of MHBs detected in cattle and human. (a) Functional enrichment of genes associated with cattle specific MHB (CMHB) and human & cattle shared MHB (HCMHB). BP: Biological Process; CC: Cellular Component. (b) Gene density (gene number per kb) in CMHBs and HCMHBs. (c) Distribution of Phastcon scores of CMHBs and HCMHBs. **Figure S3.** Characterization of highly variable methylated regions (HVMRs), hypomethylated conserved regions (Hypo CMRs) and hypermethylated conserved regions (Hyper CMRs). (a) Functional enrichment of genes associated with Hypo CMRs. KEGG: Kyoto Encyclopedia of Genes and Genomes; BP: Biological Process; CC: Cellular Component. (b) Motif enrichments of HVMRs, Hypo CMRs and Hyper CMRs. (c) Enrichment of human orthologous genes associated with HVMRs, Hypo CMRs and Hyper CMRs in tissue-specific genes. **Figure S4.** A trait-related VMR was associated with an SNP within 1 Mb distance**.** (a) The UCSC browser of the epigenetic markers associated with CCR and DPR as well as the methylation QTLs (meQTLs) associated with the trait-related VMR (chr18: 56560453–56,560,476). (b) Association studies between the meQTL (rs41893756) and 35 bovine complex traits in 27, 214 Holstein bulls. PL and SCE were the most significant traits associated with the meQTL. (c) Methylation levels of the trait-related VMR in two genotypes of rs41893756.

**Additional file 2: Table S1.** Identified methylation haplotype blocks (MHBs) in sperm DNA methylation. **Table S2.** Identified highly variable methylated regions (HVMRs), hypomethylated conserved regions (Hypo CMRs) and hypermethylated conserved regions (Hyper CMRs). **Table S3.** Significant VMRs associated with bovine complex traits and the most significant meQTL within 1 Mb of these VMRs. Some meQTL were within the reproduction-related QTLs (https://www.animalgenome.org/cgi-bin/QTLdb/BT/index). **Table S4.** Sperm sample description. **Table S5.** Statistics summary of predicted transmitting ability (PTA) values and the accuracy for Bovine complex traits in association studies with VMRs.

## Data Availability

All high-throughput sequencing data were deposited in NCBI GEO database under accession numbers GSE119263, GSE106538, and GSE131851.

## Abbreviations

BDE: Body depth
BTA: *bos taurus* autosome
CCR: Cow conception rate
CE: Calving ease
CMHB: Cattle-specific MHB
CMR: Conserved methylated region
DPR: Daughter pregnancy rate
eeQTL: Exon expression QTL
FDR: False discovery rate
geQTL: Gene expression QTL
GO: Gene ontology
GWAS: Genome wide association studies
HCMHB: Common MHB between human and cattle
HCR: Heifer conception rate
HVMR: Highly variably methylated region
hyper-CMR: Conserved hypermethylated region
hypo-CMR: Conserved hypomethylated region
LD: Linkage disequilibrium
meQTL: Methylation QTL
MHB: Methylation haplotype block
PCA: Principle component analysis
PL: Productive life
PTA: Predicted transmitting ability
EWAS: Epigenetic association studies
QTL: Quantitative trait loci
SB: Stillbirth
SCR: Sire conception rate
sQTL: Splicing QTL
ST_PL: Standard length of productive life
TFBS: Transcription factor binding sites
UTR: Untranslated region
VMR: Variably methylated region
WGBS: Whole genome bisulfite sequencing
WGCNA: Weighted correlation network analysis
