# Supplementary material for: Analyses of inter-individual variations of sperm DNA methylation and their potential implications in cattle
Source: BMC Genomics. 2019 Nov 21;20:888. doi: 10.1186/s12864-019-6228-6 (PMC6873545; doi:10.1186/s12864-019-6228-6)
Supplement: Supplementary file 1 — Additional file 1: Figure S1. Correlation among individuals. (a) Pearson correlation among individuals of known genome features. (b) Heatmap of Pearson correlation in methylation haplotype blocks (MHBs) among individuals. Figure S2. Comparison of MHBs detected in cattle and human. (a) Functional enrichment of genes associated with cattle specific MHB (CMHB) and human & cattle shared MHB (HCMHB). BP: Biological Process; CC: Cellular Component. (b) Gene density (gene number per kb) in CMHBs and HCMHBs. (c) Distribution of Phastcon scores of CMHBs and HCMHBs. Figure S3. Characterization of highly variable methylated regions (HVMRs), hypomethylated conserved regions (Hypo CMRs) and hypermethylated conserved regions (Hyper CMRs). (a) Functional enrichment of genes associated with Hypo CMRs. KEGG: Kyoto Encyclopedia of Genes and Genomes; BP: Biological Process; CC: Cellular Component. (b) Motif enrichments of HVMRs, Hypo CMRs and Hyper CMRs. (c) Enrichment of human orthologous genes associated with HVMRs, Hypo CMRs and Hyper CMRs in tissue-specific genes. Figure S4. A trait-related VMR was associated with an SNP within 1 Mb distance. (a) The UCSC browser of the epigenetic markers associated with CCR and DPR as well as the methylation QTLs (meQTLs) associated with the trait-related VMR (chr18: 56560453–56,560,476). (b) Association studies between the meQTL (rs41893756) and 35 bovine complex traits in 27, 214 Holstein bulls. PL and SCE were the most significant traits associated with the meQTL. (c) Methylation levels of the trait-related VMR in two genotypes of rs41893756. [file 12864_2019_6228_MOESM1_ESM.docx]

**Supplementary Figures**


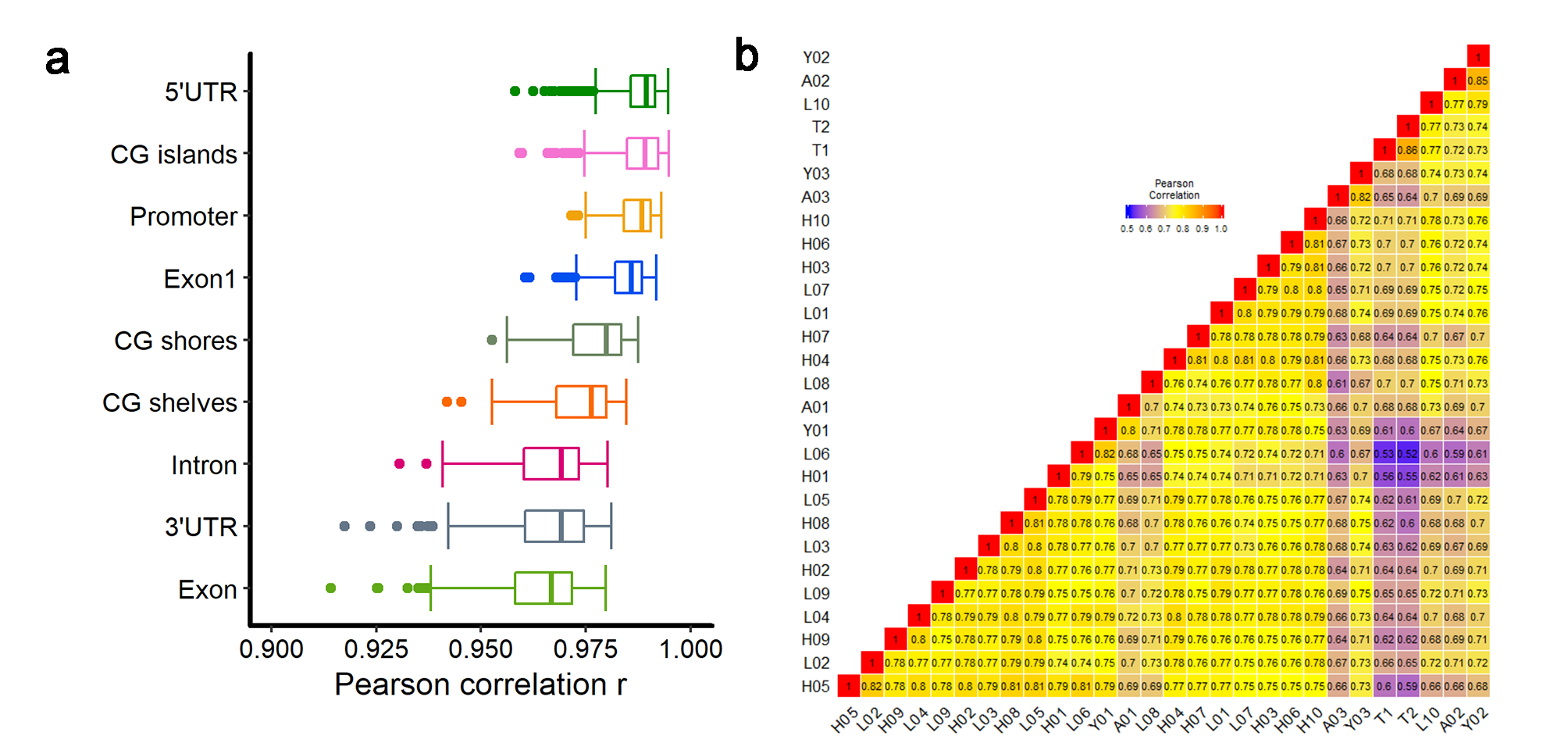


**Figure S1. Correlation among individuals.** (a) Pearson correlation among individuals of known genome features. (b) Heatmap of Pearson correlation in methylation haplotype blocks (MHBs) among individuals.


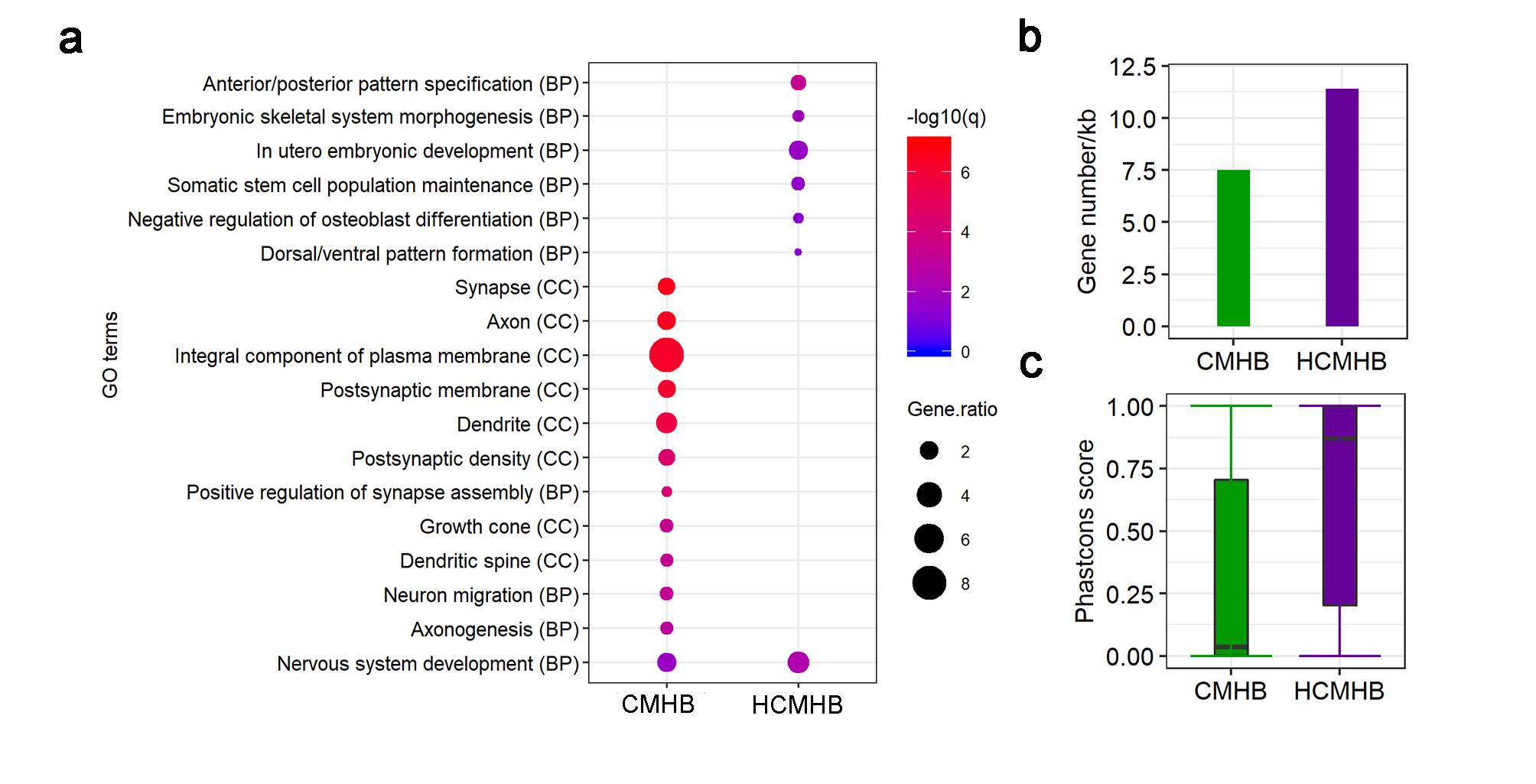


**Figure S2. Comparison of MHBs detected in cattle and human.** (a) Functional enrichment of genes associated with cattle specific MHB (CMHB) and human & cattle shared MHB (HCMHB). BP: Biological Process; CC: Cellular Component. (b) Gene density (gene number per kb) in CMHBs and HCMHBs. (c) Distribution of Phastcon scores of CMHBs and HCMHBs.


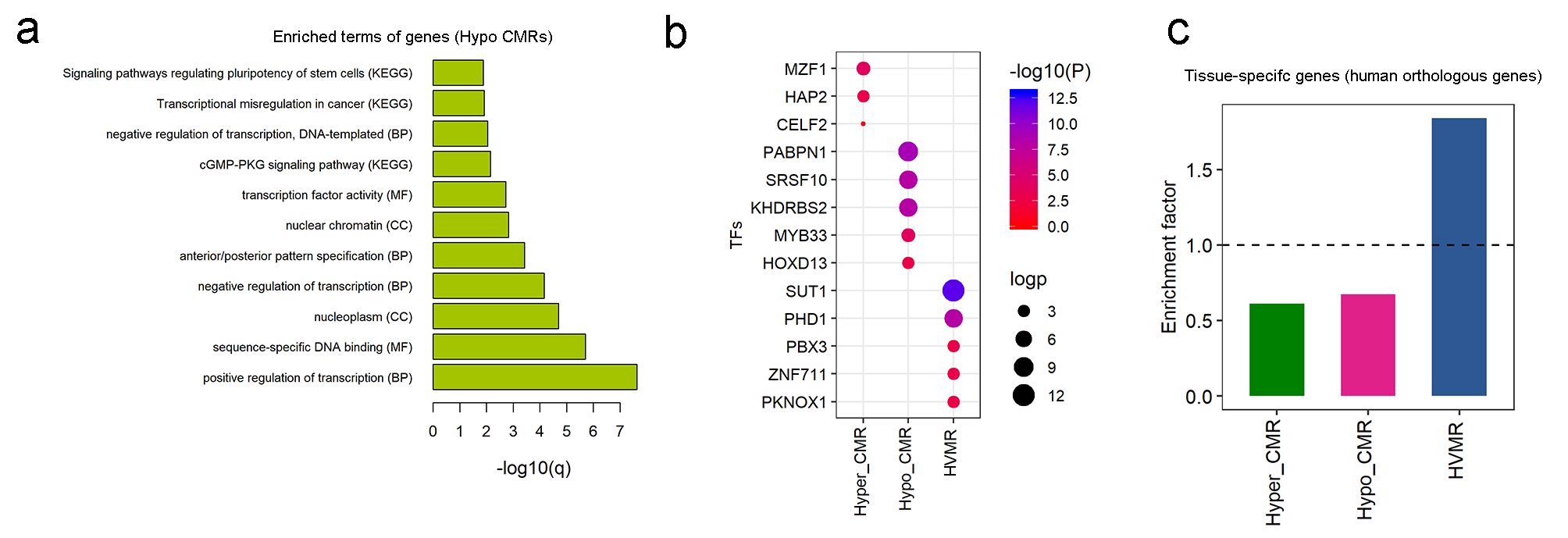


**Figure S3. Characterization of highly variable methylated regions (HVMRs), hypomethylated conserved regions (Hypo CMRs) and hypermethylated conserved regions (Hyper CMRs).** (a) Functional enrichment of genes associated with Hypo CMRs. KEGG: Kyoto Encyclopedia of Genes and Genomes; BP: Biological Process; CC: Cellular Component. (b) Motif enrichments of HVMRs, Hypo CMRs and Hyper CMRs. (c) Enrichment of human orthologous genes associated with HVMRs, Hypo CMRs and Hyper CMRs in tissue-specific genes.


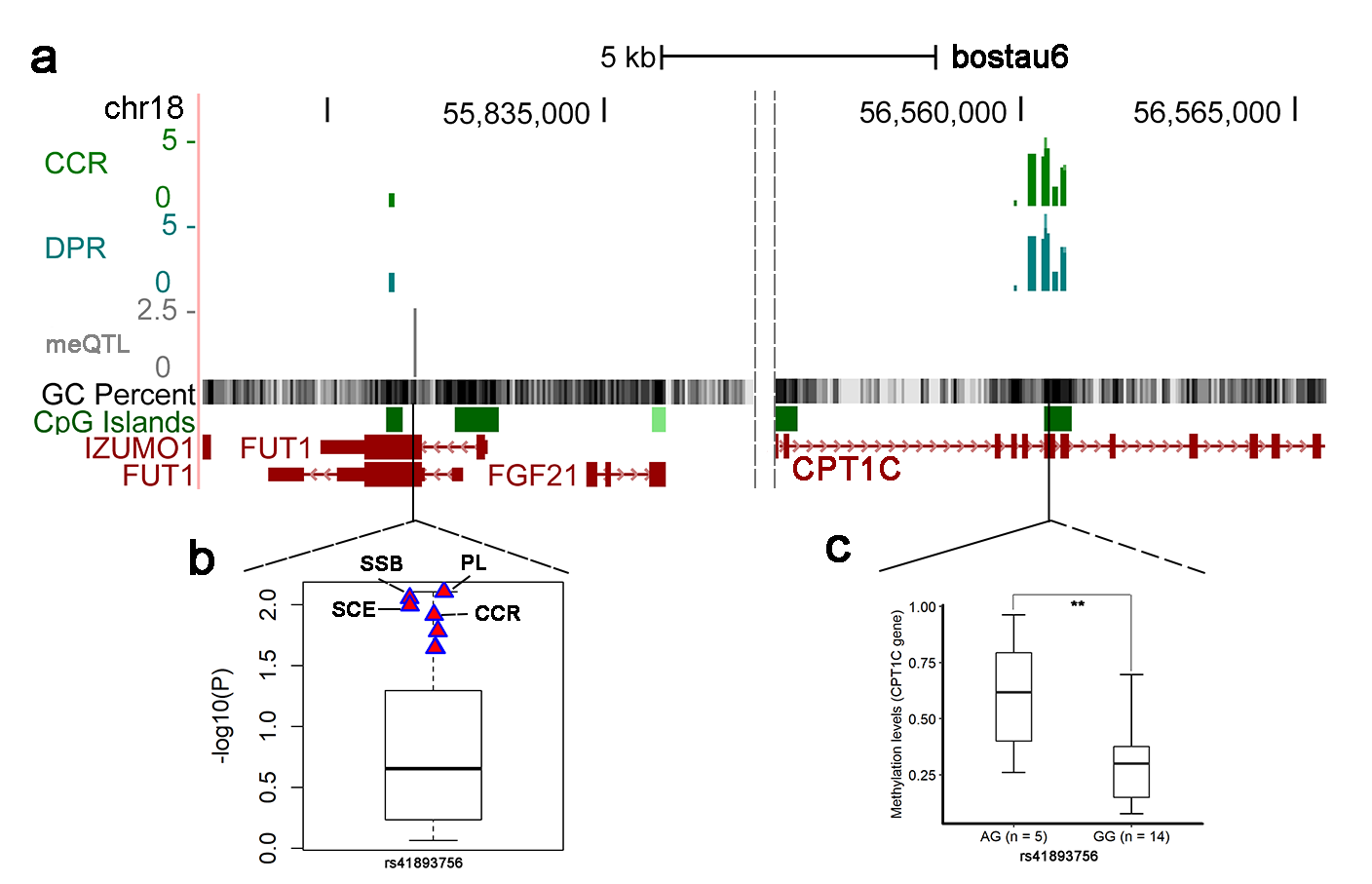


**Figure S4.** **A trait-related VMR was associated with an SNP within 1Mb distance.** (a) Ucsc browser of the epigenetic markers associated with CCR and DPR as well as the methylation QTLs (meQTLs) associated with the trait-related VMR (chr18: 56560453-56560476). (b) Association studies between the meQTL (rs41893756) and 35 bovine complex traits in 27, 214 Holstein bulls [[33](#_ENREF_33)]. PL and SCE were the most significant traits associated with the meQTL. (c) Methylation levels of the trait-related VMR in two genotypes of rs41893756.
